# Supplementary material for: Host circadian clocks do not set the schedule for the within-host replication of malaria parasites
Source: Proc Biol Sci. 2020 Aug 12;287(1932):20200347. doi: 10.1098/rspb.2020.0347 (PMC7575513; doi:10.1098/rspb.2020.0347)
Supplement: Electronic Supplementary Information [file rspb20200347supp1.docx]

**ELECTRONIC SUPPLEMENTARY MATERIAL**

**METHODOLOGY**

***Statistical Analysis***

Time of onset of locomotor activity and overall period estimates for locomotor activity and body temperature data were calculated using Clocklab (Actimetrics). Harmonic regression was performed on parasite data using CircWave (v. 1.4, courtesy of R. Hut; <http://www.euclock.org>) with a fixed period of 24h and an alpha of 0.05. All other statistical analyses were carried out using R version 3.5.0. A second periodicity analysis was also performed using the package JTK_CYCLE with a fixed period of 24h [1]. Correlations between feeding events and locomotor activity was tested using a generalised linear model with a zero-inflated poisson regression (package pscl) and activity measures were analysed using linear mixed-effects models with mouse fitted as a random effect. Output model fit measures for amplitude and phase, as well disease severity (red blood cell loss) and parasite performance (maximum parasite density) measures, were compared between groups using linear models and models were confirmed for best fit by comparing AICC values. Parasite IDC data from three *Per1/2*-null mice, across both experiments (experiment 1: 1x *ad lib* group; experiment 2: 1x *ad lib* group and 1x TRF group), were excluded from the final reporting of amplitude and phase due to a lack of significant rhythmicity (Table S1). All models met model assumptions: independence of data points, normality of residuals and homogeneity of variances (confirmed through assessing the model plots, the Shapiro–Wilk test and Bartlett’s test). Whilst some variation in parasite density was observed across treatment groups, any underlying differences in replication rate are not large enough to introduce biases associated with estimating synchrony from IDC stage proportion data [2]. Mean effect sizes were calculated using bootstrapped coupled estimation (package dabestr). Measures of red blood cell loss were calculated by taking the maximum RBC value across the time series and subtracting the minimum RBC value. This minimised any issues associated with comparing data for the same GMT across groups in which host and parasite rhythms may be phased differently. For measures of circadian phase, Bayesian circular GLM’s (selected using DIC) were used (package circglmbayes), which allows a circular outcome to be regressed onto linear and categorical predictors.

***Locomotor and internal body temperature data collection***

We measured locomotor activity and internal body temperature before and during infection without disturbance to the animals using the Actual Home Cage Analysis system (Actual HCA, Actual Analytics Ltd, Edinburgh, Scotland) [3]. Specifically, Biotherm13 RFID tags (radio frequency identification; Biomark, Idaho, USA) were injected subcutaneously and both positional and temperature readings were recorded every 10ms (and then summed into 1 minute bins) via an array of antennas (spaced ~11cm apart) under each cage. To determine if locomotor activity can be used as a good proxy for feeding events, two groups of five uninfected WT and *Per1/2*-null mice were group housed (separated by genotype) in DD and filmed for 24 hours. Feeding bouts were classified from observing mice nibbling on food pellets from the hopper. These data were summed to 15 minute bins and compared with locomotor activity data obtained from RFID tags and the Actual Home Cage Analysis system.

**RESULTS**

***Assumptions of the experimental designs***

*Rhythms in locomotor activity*

Only wild type mice exhibited daily periodicity in locomotor activity with a mean period of 23.96 (± 0.01 SEM) for *ad lib* fed and 23.82 (± 0.03 SEM) for TRF fed (Figure S1; Figure S2). WT *ad lib* fed mice had an average median onset of activity per day at 20.1 (±0.21 SEM; GMT) while TRF WT mice started their activity 3 hours earlier at 17.2 (±0.54 SEM; GMT; χ^2^_1_= 9.71, *p* = 0.002). The level of activity during rest periods compared to active periods in WT mice was not influenced by feeding regime (χ^2^_1_= 0.06, *p* = 0.81). *Per1/2*-null mice did not exhibit rhythmic locomotor activity (i.e. are behaviourally arrhythmic) as evident from actograms (Figure S1), lomb-scargle periodograms (Figure S2) and 24 hour activity profile (Figure S3a). Total activity per 24h period was 41% lower in *Per1/2*-null mice compared to WT mice (mean transitions ±SEM: WT = 1636 ±70.7, *Per1/2*-null = 958 ±48.5; χ^2^_1_ = 7.29, *p* < 0.001) and activity was not influenced by feeding regime (χ^2^_1_ = 0.05, *p* = 0.83) or its interaction with genotype (χ^2^_1_ = 0.002, *p* = 0.97).

*Rhythms in internal body temperature*

Both WT TRF and *ad lib* mice exhibited rhythms in internal body temperature (becoming ~1°C cooler during rest periods; Figure S1 & Figure S3b) with periods close to 24 hours (mean period ± SEM: WT *ad lib* = 24.00 ± 0, WT TRF = 23.91 ± 0.04). Unlike locomotor activity, *Per1/2*-null TRF mice exhibited rhythmicity in body temperature with a mean period of 23.98 (±0.01 SEM), becoming, ~2°C cooler during periods when food was unavailable. No such temperature rhythms were observed in *ad lib* fed *Per1/2*-null mice (Figure S1; Figure S2; Figure S3b).

*Locomotor activity as a proxy for feeding activity*

Finally, the experimental designs required that locomotor activity was strongly correlated with feeding events in both WT mice and *Per1/2*-null *ad lib* fed mice, which was the case (Zero-inflated count model; WT: z = 21.22, *p* < 0.001, *Per1/2*-null: z = 16.71, *p* < 0.001; Figure S4). Non-feeding events were also correlated with zero activity (Zero-inflated binomial model; WT: z = -2.54, *p* = 0.011, *Per1/2*-null: ZeroInfl binomial model: z = -2.770, *p* = 0.006). Thus, if animals were moving they generally were also eating, and therefore we use locomotor activity as a proxy for feeding events during periods when food was available.

**
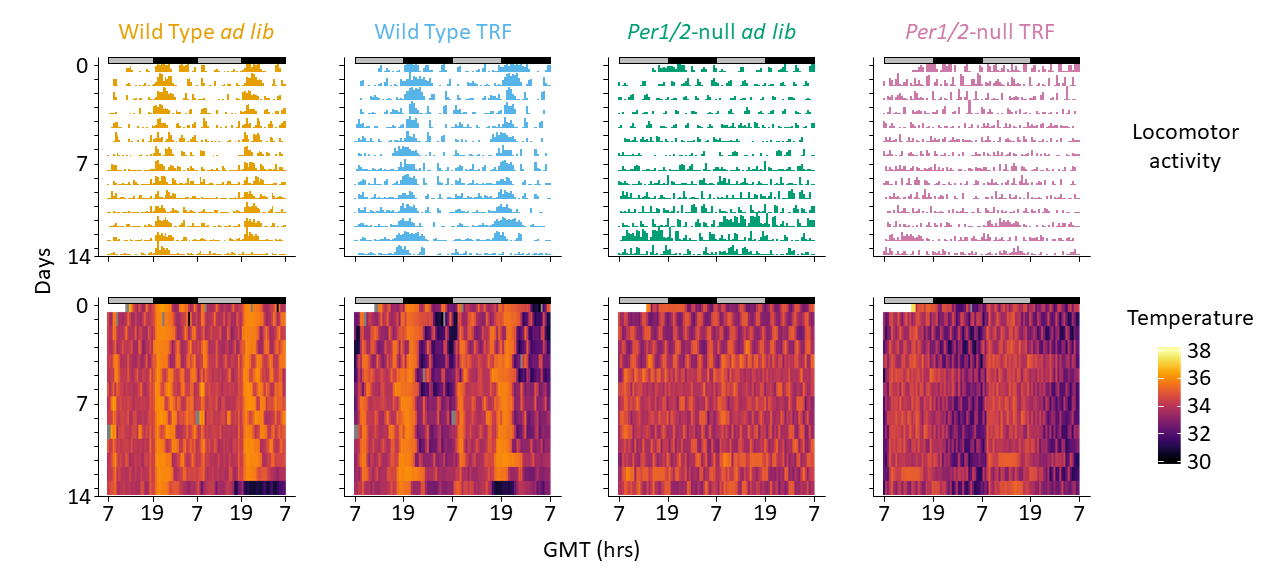
**

**Figure S1**

**Locomotor activity and internal body temperature.** Locomotor activity (top) and internal body temperature (bottom) measures from ‘representative mice’ recorded via subcutaneous RFID chips and an antennae array over 14 days pre-infection (summed into 5 minute bins and double plotted). Light and grey horizontal bars represent subjective day and night, respectively. WT and *Per1/2*-null mice were given food either *ad lib* or had access to food restricted to 10 hours each day between 09:00 and 19:00 (time restricted feeding, TRF).

**
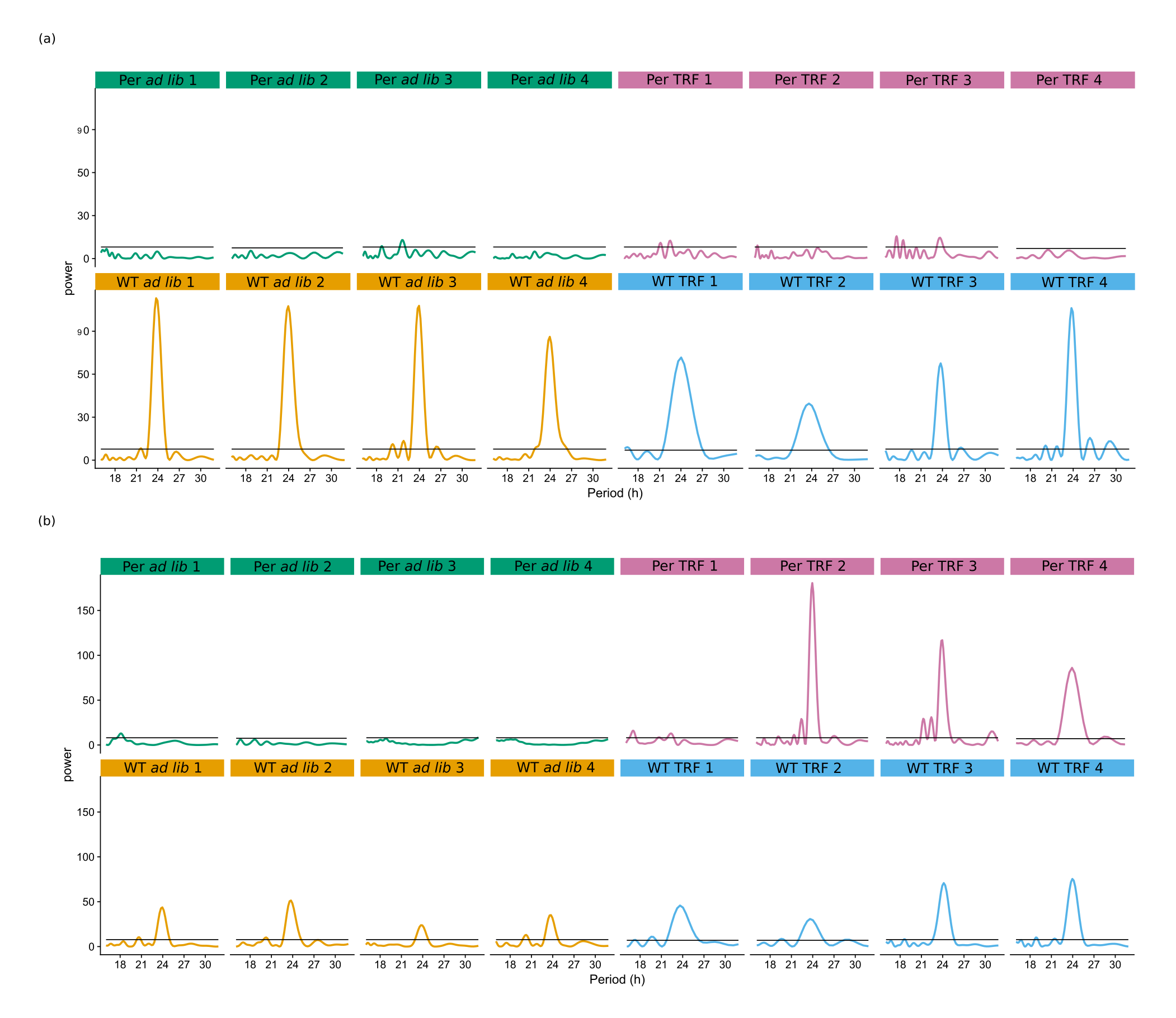
**

**Figure S2**

**Lomb-scargle periodograms for (a) locomotor activity and (b) body temperature.** Metrics plotted for four ‘representative mice’ per treatment recorded via subcutaneous RFID chips and an antennae array over 14 days pre-infection (summed into 5 minute bins). Dark horizontal line indicates a significance threshold of 0.01. Wild type (WT) and *Per1/2*-null (Per) mice were given food either *ad lib* diet or had access to food restricted to 10 hours per day (time restricted feeding, TRF).


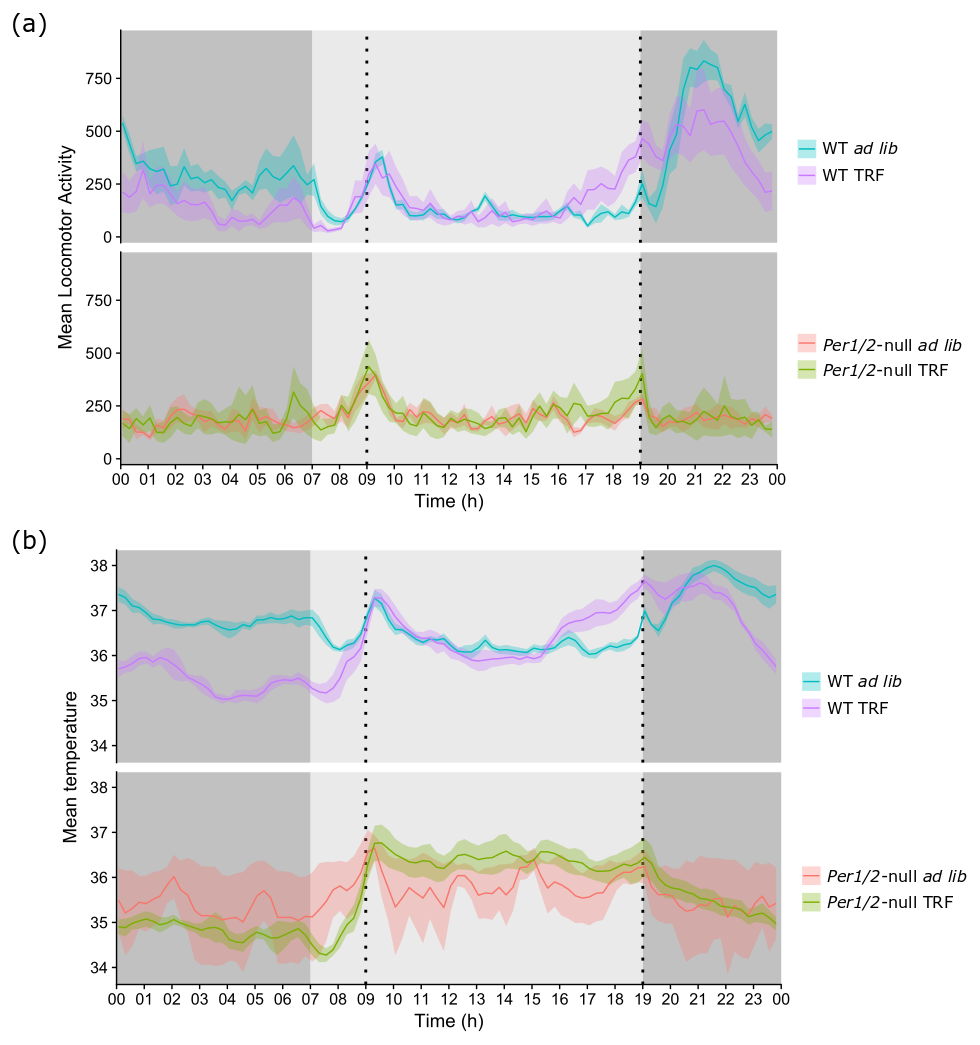


**Figure S3:**

**Average 24h profiles for (a) locomotor activity and (b) internal body temperature** Mean (± SEM) from four ‘representative’ mice in each treatment group recorded via subcutaneous RFID chips over 14 days pre-infection (summed (a) or averaged (b) into 15 minute bins). Light and grey horizontal bars represent subjective day and night, respectively. Wild type (WT) and *Per1/2*-null mice were given food either *ad lib* or had access to food restricted to 10 hours each day between 09:00 and 19:00 (time restricted feeding, TRF). Dotted lines represent food in (left) and food removed (right) for TRF treatments.


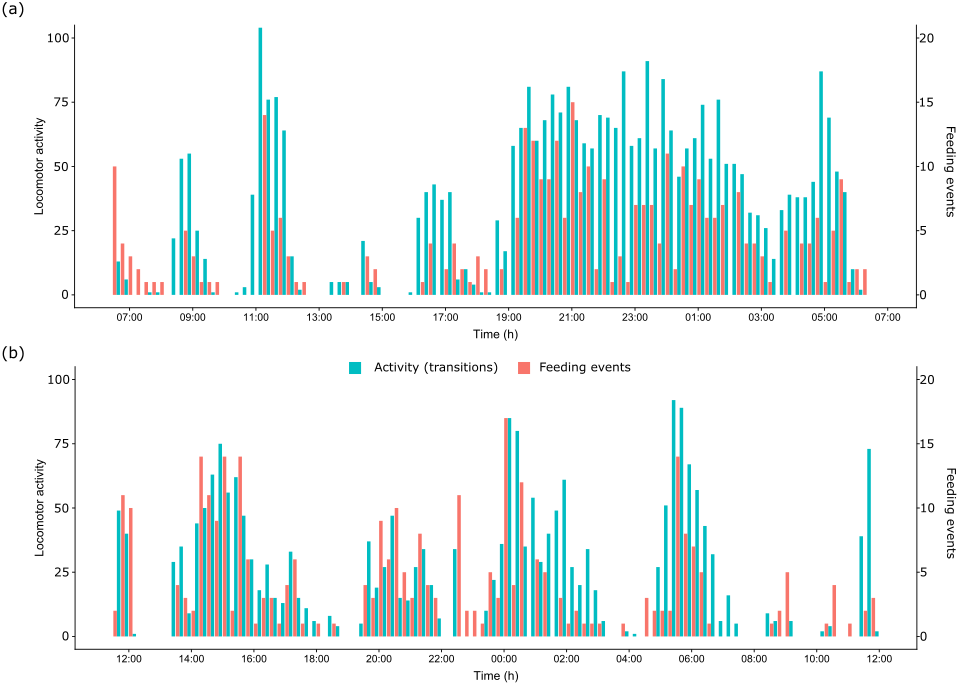


**Figure S4**

**Locomotor activity and feeding bouts for Wild type (a) and *Per1/2*-null (b) mice across one 24h period.** Locomotor activity is recorded via subcutaneous RFID chip via an antennae array, and feeding bouts (mice eating pelleted food from the food hopper) were tallied from video footage (summed in 15 minute bins). Mice are group housed (n=5) and the sum recordings from all individuals in a cage are plotted for each bin.

**
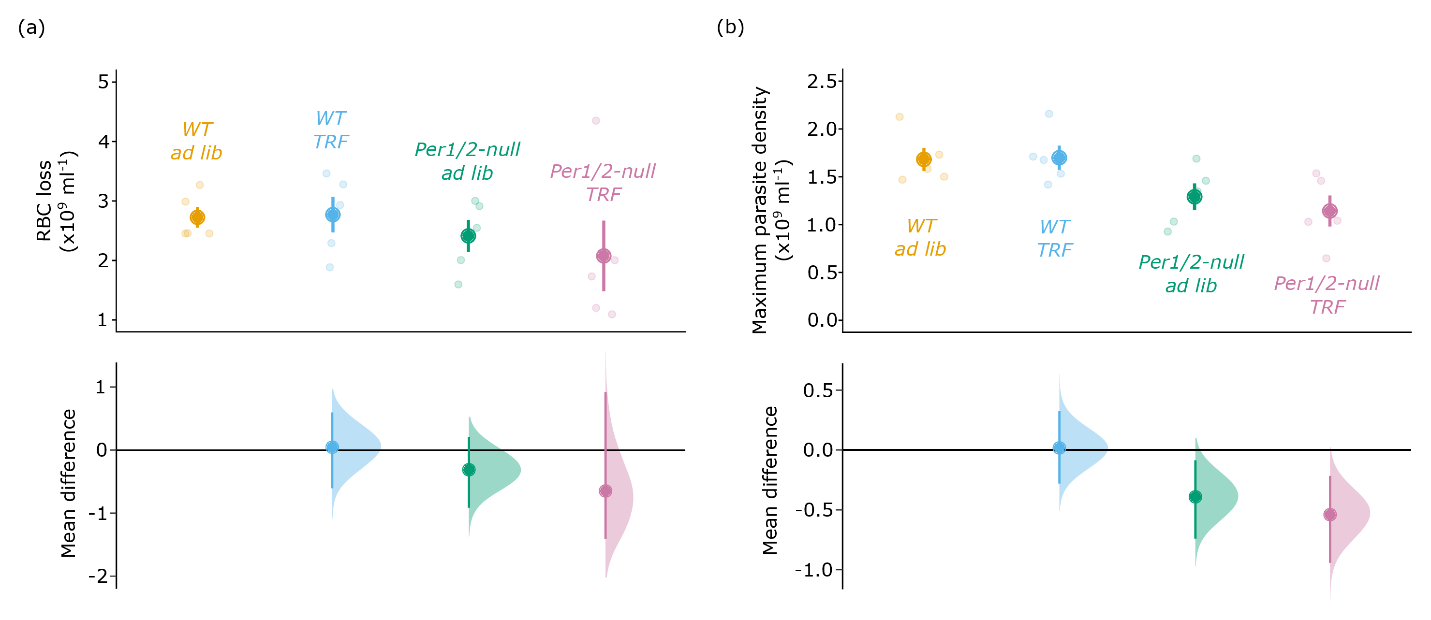
**

**Figure S5**

**Virulence and performance of infections initiated with desynchronised parasites.** (a) Virulence as measured by mean (±SEM) red blood cell (RBC) loss across infections (day 5-6 PI) in wild type and *Per1/2*-null mice, according to feeding regime. (b) Parasite performance as measured by mean (±SEM) maximum parasite density. Behind each mean, individual infections are shown as lighter points. Effect sizes relative to ‘WT *ad lib*’ group are plotted on the lower axes as a bootstrap sampling distribution (mean difference ± 95% CI depicted as a point with error bars). WT and *Per1/2*-null mice were given food either *ad lib* diet or had access to food restricted to 10 hours per day (time restricted feeding, TRF). n=5 for WT and TRF groups, n = 4 for *Per1/2*-null *ad lib* group)


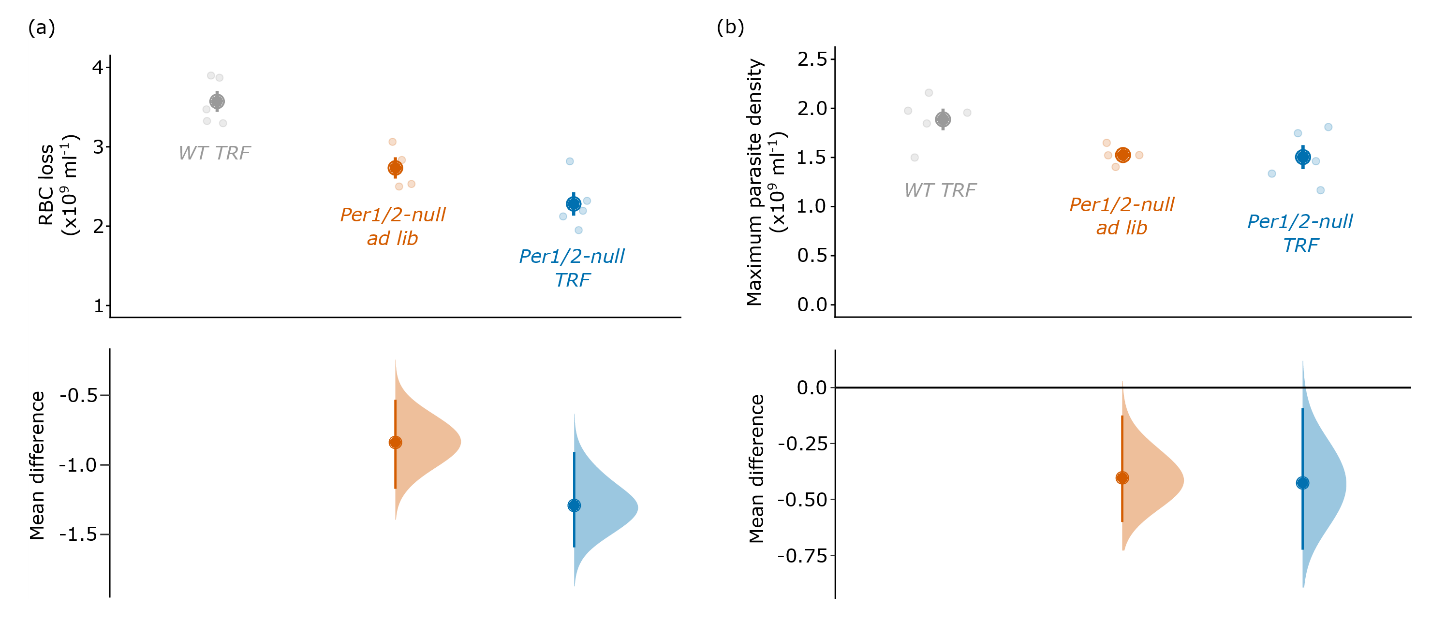


**Figure S6**

**Virulence and performance of infections initiated with synchronous parasites.** (a) Virulence as measured by mean (±SEM) red blood cell (RBC) loss across infections (day 5-6 PI) in wild type and *Per1/2*-null mice, according to feeding regime. (b) Parasite performance as measured by mean (±SEM) maximum parasite density. Behind each mean, individual infections are shown as lighter points. Effect sizes relative to ‘WT TRF’ group are plotted on the lower axes as a bootstrap sampling distribution (mean difference ± 95% CI depicted as a point with error bars). WT and *Per1/2*-null mice were given food either *ad lib* diet or had access to food restricted to 10 hours per day (time restricted feeding, TRF). n = 5 for WT and *Per1/2*-null TRF, n = 4 for *Per1/2*-null *ad lib* groups.

|  | **Group** | **Mouse** | **Constant** | **Sin** | **Cos** | **R^2^** | **F-stat** | **P value** |
| --- | --- | --- | --- | --- | --- | --- | --- | --- |
| Expt. 1 | WT *ad lib* | M1 | 0.573 | 0.3879 | 0.0612 | 0.87 | 19.79 | 0.002 |
|  |  | M2 | 0.478 | 0.2159 | -0.2405 | 0.82 | 13.98 | 0.006 |
|  |  | M3 | 0.519 | 0.3695 | -0.1649 | 0.90 | 26.23 | 0.001 |
|  |  | M4 | 0.513 | 0.3454 | -0.1078 | 0.77 | 9.97 | 0.012 |
|  |  | M5 | 0.598 | 0.3914 | 0.0002 | 0.89 | 25.49 | 0.001 |
|  |  |  |  |  |  |  |  |  |
|  | WT TRF | M1 | 0.547 | 0.1045 | 0.4041 | 0.90 | 26.01 | 0.001 |
|  |  | M2 | 0.558 | -0.0105 | 0.2394 | 0.84 | 16.12 | 0.004 |
|  |  | M3 | 0.518 | -0.0431 | 0.2374 | 0.86 | 19.03 | 0.003 |
|  |  | M4 | 0.516 | -0.1116 | 0.2312 | 0.98 | 135.26 | <0.001 |
|  |  | M5 | 0.509 | -0.1386 | 0.2784 | 0.96 | 70.41 | <0.001 |
|  |  |  |  |  |  |  |  |  |
|  | *Per1/2*-null *ad lib* | M1 | 0.467 | -0.1052 | -0.0678 | 0.60 | 4.50 | *NS* |
|  |  | M2 | 0.482 | -0.1095 | -0.0137 | 0.73 | 8.04 | 0.020 |
|  |  | M3 | 0.507 | -0.1246 | 0.1375 | 0.87 | 20.68 | 0.002 |
|  |  | M4 | 0.483 | -0.0405 | 0.3156 | 0.83 | 14.80 | 0.005 |
|  |  | M5 | 0.479 | -0.1661 | -0.1066 | 0.85 | 17.19 | 0.003 |
|  |  |  |  |  |  |  |  |  |
|  | Per1/2-null TRF | M1 | 0.527 | 0.0759 | 0.3174 | 0.78 | 10.81 | 0.010 |
|  |  | M2 | 0.443 | -0.4742 | 0.1685 | 0.91 | 31.78 | <0.001 |
|  |  | M3 | 0.469 | -0.4462 | 0.0896 | 0.96 | 63.73 | <0.001 |
|  |  | M4 | 0.538 | -0.434 | 0.2533 | 0.98 | 119.33 | <0.001 |
|  |  | M5 | 0.482 | -0.1423 | 0.3075 | 0.73 | 8.31 | 0.0186 |
|  |  |  |  |  |  |  |  |  |
| Expt. 2 | WT TRF | M1 | 0.544 | -0.1761 | 0.4480 | 0.92 | 34.83 | <0.001 |
|  |  | M2 | 0.587 | 0.0808 | 0.5314 | 0.90 | 27.50 | 0.001 |
|  |  | M3 | 0.555 | -0.1773 | 0.4640 | 0.94 | 44.41 | <0.001 |
|  |  | M4 | 0.511 | -0.198 | 0.3970 | 0.93 | 38.90 | <0.001 |
|  |  | M5 | 0.508 | -0.1495 | 0.4129 | 0.90 | 26.38 | 0.001 |
|  |  |  |  |  |  |  |  |  |
|  | *Per1/2*-null *ad lib* | M1 | 0.545 | -0.2375 | 0.0212 | 0.61 | 4.79 | *NS* |
|  |  | M2 | 0.573 | -0.1688 | -0.0752 | 0.73 | 7.93 | 0.021 |
|  |  | M3 | 0.566 | -0.1865 | 0.0083 | 0.68 | 6.29 | 0.034 |
|  |  | M4 | 0.590 | -0.219 | 0.0235 | 0.77 | 9.84 | 0.013 |
|  |  |  |  |  |  |  |  |  |
|  | Per1/2-null TRF | M1 | 0.507 | -0.0306 | 0.4395 | 0.89 | 23.45 | 0.002 |
|  |  | M2 | 0.601 | -0.1695 | 0.3972 | 0.90 | 27.15 | 0.001 |
|  |  | M3 | 0.555 | -0.0106 | 0.4674 | 0.95 | 53.23 | <0.001 |
|  |  | M4 | 0.565 | -0.2302 | 0.3863 | 0.74 | 8.66 | 0.017 |
|  |  | M5 | 0.580 | 0.045 | 0.3182 | 0.59 | 4.38 | *NS* |
|  |  |  |  |  |  |  |  |  |

**Table S1**

**Mouse model fits from F tested forward linear harmonic regression. Periodicity model fits for each infection using Circwave. NS indicates fit did not meet the alpha of 0.05. Period (*Tau*) was fixed to 24 hours.**

|  | Group | JTK Amplitude [95% CI] | JTK Amplitude P value | JTK Phase (hrs) | Benjamani-Hochberg q-value | adj. P value |
| --- | --- | --- | --- | --- | --- | --- |
| Expt. 1 | WT *ad lib* | 0.65 [0.39,0.88] | < 0.001 | 8 | 7.5E-09 | <0.0001 |
|  | WT TRF | 0.53 [0.37,0.70] | < 0.001 | 24 | 2.7E-11 | <0.0001 |
|  | Per1/2-null *ad lib* | 0.27 [0.10, 0.40] | 0.003 | 20 | 1.3E-03 | 0.001 |
|  | Per1/2-null TRF | 0.86 [0.61,0.99] | < 0.001 | 20 | 8.3E-08 | <0.0001 |
|  |  |  |  |  |  |  |
| Expt. 2 | WT TRF | 0.97 [0.58,1.07] | < 0.001 | 24 | 6.4E-13 | <0.0001 |
|  | Per1/2-null *ad lib* | 0.30 [0.18, 0.48] | 0.0010 | 18 | 6.5E-05 | <0.0001 |
|  | Per1/2-null TRF | 0.84 [0.67, 1.03] | < 0.001 | 24 | 7.5E-09 | <0.0001 |

**Table S2**

**Periodicity analysis for infections using the non-parametric periodicity test JTK_Cycle.** Mice were grouped by genotype and feeding regime (n = 5, except *Per1/2*-null *ad lib* for which n = 4) and period (*Tau*) was fixed to 24 hours.

|  | model | DIC | ΔDIC |
| --- | --- | --- | --- |
| Expt. 1 | **phase ~ genotype:feeding_regime** | **40.27** | **27.84** |
|  | phase ~ genotype + feeding_regime | 55.37 | 12.74 |
|  | phase ~ genotype | 59.93 | 8.18 |
|  | phase ~ feeding_regime | 64.32 | 3.79 |
|  | phase ~ 1 | 68.11 | - |
|  |  |  |  |
| Expt. 2 | **phase ~ feeding_regime** | **4.07** | **23.78** |
|  | phase ~ genotype + treatment | 6.32 | 21.53 |
|  | phase ~ genotype | 27.49 | 0.36 |
|  | phase ~ 1 | 27.85 | - |

**Table S3 Differences in DIC (ΔDIC) between Bayesian circular generalized linear models explaining parasite phase. Minimum model in bold. Parasite phase is best explained by a mouse genotype:feeding_regime interaction in Experiment 1 and by feeding regime alone in Experiment 2.**

|  | Group | Mean Amplitude difference  (95% CI) | Mean Phase difference (hours) (95% CI) | Mean RBC loss difference (x10^8^ ml^-1^) (95% CI) | Mean max parasite density difference (x10^8^ ml^-1^) (95% CI) |
| --- | --- | --- | --- | --- | --- |
| **Expt. 1** | WT *ad lib*  (reference group) | Reference group | Reference group | Reference group | Reference group |
|  | WT TRF | -0.16  (-0.26, 0.00) | **-7.74  (-9.26, -6.30)** | 0.05  (-0.61 0.60) | 0.17  (-2.81, 3.25) |
|  | *Per1/2*-null *ad lib* | **-0.34  (-0.48, -0.17)** | **-11.6  (-14.7, -8.31)** | -0.31  (-0.92, 0.21) | -3.91  (-7.41, 0.87) |
|  | *Per1/2*-null TRF | 0.10  (-0.06, 0.23) | **-10.1  (-12.1, -7.21)** | -0.65  (-1.41, 0.92) | **-5.40  (-9.42, -2.17**) |
|  |  |  |  |  |  |
| **Expt. 2** | WT TRF  (reference group) | Reference group | Reference group | Reference group | Reference group |
|  | *Per1/2*-null *ad lib* | **-0.565  (-0.64, -0.50)** | **-5.26  (-6.84, -4.23**) | **-0.84  (-1.17, -0.53)** | **0.04  (-0.06, -0.01)** |
|  | *Per1/2*-null TRF | -0.065  (-0.14, -0.00) | -0.09  (-1.19, 0.99) | **-1.29  (-1.59, -0.91)** | **0.04  (-0.07, -0.01)** |

**Table S4**

**Mean effect sizes (with bias-corrected 95% confidence intervals) relative to control infections.** Mean effect sizes calculated using 5000 bootstrap resamples and are differences relative to ‘WT *ad lib*’ mice for Experiment 1 and ‘WT TRF’ mice for Experiment 2. In bold are effects in which the 95% confidence intervals do not include zero.

|  | Group | Mean starting  weight ±SEM  (g) | Mean ending  weight ±SEM  (g) | Mean  % weight loss | Mean difference in % weight loss  (95% CI) |  |
| --- | --- | --- | --- | --- | --- | --- |
| **Expt. 1** | WT *ad lib* | 24.9 ± 0.6 | 24.0 ± 0.2 | 3.4 ± 1.8 | Reference group |  |
|  | WT TRF | 28.2 ± 0.5 | 25.6 ± 0.6 | 9.2 ± 2.2 | 5.9 (-0.9, 9.8) |  |
|  | *Per1/2*-null *ad lib* | 29.5 ± 1.0 | 26.0 ± 0.3 | 11.5 ± 3.2 | **8.1 (2.7, 15.6)** |  |
|  | *Per1/2*-null TRF | 24.8 ± 0.6 | 23.1 ± 0.4 | 6.7 ± 1.9 | 3.3 (-1.5, 7.5) |  |
|  |  |  |  |  |  |  |
| **Expt. 2** | WT TRF | 27.5 ± 0.6 | 25.5 ± 0.4 | 7.3 ± 1.3 | Reference group |  |
|  | *Per1/2*-null *ad lib* | 23.7 ± 0.7 | 21.5 ± 0.6 | 9.2 ± 2.4 | 1.9 (-1.7, 7.8) |  |
|  | *Per1/2*-null *TRF* | 23.5 ± 0.3 | 20.7 ± 0.4 | 12.0 ± 1.4 | **4.7 (1.6, 8.2)** |  |

**Table S5**

**Mean mouse weights and weight loss.** WT and *Per1/2*-null mice were given food either *ad lib* or had access to food restricted to 10 hours per day between 09:00 and 19:00 (time restricted diet, TRF). Starting measures were taken at Day 2 PI and ending weights at Day 6 PI. Mean effect sizes calculated using 5000 bootstrap resamples and are differences relative to reference groups: ‘WT *ad lib*’ mice for Experiment 1 and ‘WT TRF’ mice for Experiment 2. In bold are effects in which the 95% confidence intervals do not include zero.

**REFERENCES**

1. Hughes M.E., Hogenesch J.B., Kornacker K. 2010 JTK_CYCLE: an efficient nonparametric algorithm for detecting rhythmic components in genome-scale data sets. *J Biol Rhythms* **25**(5), 372-380.

2. Greischar M.A., Reece S.E., Savill N.J., Mideo N. 2019 The Challenge of Quantifying Synchrony in Malaria Parasites. *Trends Parasitol*.

3. Bains R.S., Cater H.L., Sillito R.R., Chartsias A., Sneddon D., Concas D., Keskivali-Bond P., Lukins T.C., Wells S., Arozena A.A., et al. 2016 Analysis of Individual Mouse Activity in Group Housed Animals of Different Inbred Strains Using a Novel Automated Home Cage Analysis System. *Front Behav Neurosci* **10**.
